# Supplementary material for: Perceiving threat in others: The role of body morphology
Source: PLoS One. 2021 Apr 8;16(4):e0249782. doi: 10.1371/journal.pone.0249782 (PMC8031394; doi:10.1371/journal.pone.0249782)
Supplement: S1 Table — (DOCX) [file pone.0249782.s001.docx]

**S1 Table.** **Breakdown of the dimensions of the Daz human male body stimuli (in centimetres), varying by 7 levels of musculature.**

|  | **Below Knee Circ.** | **Ankle Circ.** | **Knee to Ankle** | **Inseam** | **Thigh Circ.** | **Crotch to Knee** | **Low Hip Circ.** | **Wrist Circ.** | **Elbow to Wrist** | **Forearm Circ.** | **Shoulder to Elbow** | **Bicep Circ.** | **Shoulder to Wrist** | **Bust Circ.** | **Waist Circ.** | **Waist to Thigh** | **Shoulder to Shoulder** | **Thigh to Floor** | **Collar Bone Neck to Floor** |
| --- | --- | --- | --- | --- | --- | --- | --- | --- | --- | --- | --- | --- | --- | --- | --- | --- | --- | --- | --- |
| **Muscle**  **1** | 33.49 | 23.19 | 42.60 | 78.04 | 56.31 | 35.44 | 93.58 | 17.78 | 26.35 | 27.68 | 30.57 | 31.70 | 56.92 | 101.93 | 83.24 | 23.53 | 37.22 | 81.14 | 152.21 |
| **Muscle**  **2** | 33.86 | 23.30 | 42.59 | 78.03 | 57.70 | 35.45 | 94.76 | 17.97 | 26.37 | 28.48 | 30.82 | 33.56 | 57.19 | 104.44 | 84.27 | 23.54 | 37.08 | 81.20 | 152.27 |
| **Muscle**  **3** | 34.25 | 23.40 | 42.57 | 78.03 | 59.09 | 35.46 | 95.98 | 18.15 | 26.40 | 29.34 | 31.07 | 35.47 | 57.47 | 106.95 | 85.34 | 23.55 | 36.94 | 81.27 | 152.33 |
| **Muscle**  **4** | 34.65 | 23.52 | 42.55 | 78.02 | 60.50 | 35.47 | 97.25 | 18.34 | 26.43 | 30.24 | 31.31 | 37.43 | 57.47 | 109.45 | 86.43 | 23.56 | 36.80 | 81.34 | 152.39 |
| **Muscle**  **5** | 35.06 | 23.63 | 42.54 | 78.02 | 61.92 | 35.48 | 98.56 | 18.53 | 26.46 | 31.20 | 31.56 | 39.42 | 58.02 | 111.94 | 87.54 | 23.57 | 36.66 | 81.41 | 152.45 |
| **Muscle**  **6** | 35.49 | 23.75 | 42.52 | 78.01 | 63.34 | 35.49 | 99.90 | 18.72 | 26.50 | 32.19 | 31.80 | 41.44 | 58.29 | 114.43 | 88.66 | 23.59 | 36.53 | 81.48 | 152.51 |
| **Muscle**  **7** | 35.92 | 23.87 | 42.51 | 78.01 | 64.78 | 35.50 | 101.27 | 18.91 | 26.54 | 33.21 | 32.05 | 43.51 | 58.59 | 116.92 | 89.79 | 23.61 | 36.40 | 81.54 | 152.57 |
