# Supplementary material for: Perceiving threat in others: The role of body morphology
Source: PLoS One. 2021 Apr 8;16(4):e0249782. doi: 10.1371/journal.pone.0249782 (PMC8031394; doi:10.1371/journal.pone.0249782)
Supplement: S2 Table — (DOCX) [file pone.0249782.s002.docx]

**S2 Table. Breakdown of the dimensions of the Daz human male body stimuli (in centimetres), varying by 7 levels of emaciation.**

|  | **Below Knee Circ.** | **Ankle Circ.** | **Knee to Ankle** | **Inseam** | **Thigh Circ.** | **Crotch to Knee** | **Low Hip Circ.** | **Wrist Circ.** | **Elbow to Wrist** | **Forearm Circ.** | **Shoulder to Elbow** | **Bicep Circ.** | **Shoulder to Wrist** | **Bust Circ.** | **Waist Circ.** | **Waist to Thigh** | **Shoulder to Shoulder** | **Thigh to Floor** | **Collar Bone Neck to Floor** |
| --- | --- | --- | --- | --- | --- | --- | --- | --- | --- | --- | --- | --- | --- | --- | --- | --- | --- | --- | --- |
| **Emac 1** | 33.49 | 23.19 | 42.6 | 78.04 | 56.31 | 35.44 | 93.58 | 17.78 | 26.35 | 27.68 | 30.57 | 31.70 | 56.92 | 101.93 | 83.24 | 23.53 | 37.22 | 81.14 | 152.21 |
| **Emac 2** | 32.96 | 23.09 | 42.62 | 78.05 | 55.08 | 35.44 | 92.43 | 17.56 | 26.34 | 27.10 | 30.50 | 30.87 | 56.84 | 100.63 | 82.51 | 23.51 | 37.18 | 81.14 | 152.18 |
| **Emac 3** | 32.44 | 22.99 | 42.63 | 78.07 | 53.87 | 35.44 | 91.30 | 17.35 | 26.34 | 26.52 | 30.42 | 30.06 | 56.76 | 99.34 | 81.81 | 23.50 | 37.14 | 81.13 | 152.16 |
| **Emac 4** | 31.92 | 22.89 | 42.64 | 78.08 | 52.67 | 35.44 | 90.18 | 17.14 | 26.33 | 25.95 | 30.35 | 29.26 | 56.68 | 98.06 | 81.15 | 23.48 | 37.10 | 81.13 | 152.14 |
| **Emac 5** | 31.41 | 22.80 | 42.65 | 78.09 | 51.49 | 35.44 | 89.08 | 16.93 | 26.33 | 25.39 | 30.27 | 28.46 | 56.6 | 96.79 | 80.51 | 23.47 | 37.05 | 81.13 | 152.11 |
| **Emac 6** | 30.90 | 22.70 | 42.66 | 78.10 | 50.33 | 35.44 | 87.99 | 16.73 | 26.32 | 24.85 | 30.19 | 27.68 | 56.51 | 95.54 | 79.90 | 23.45 | 37.01 | 81.13 | 152.03 |
| **Emac 7** | 30.40 | 22.61 | 42.67 | 78.11 | 49.18 | 35.44 | 86.91 | 16.54 | 26.32 | 24.32 | 30.12 | 26.92 | 56.43 | 94.30 | 79.32 | 23.44 | 36.96 | 81.13 | 152.06 |
