# Supplementary material for: Perceiving threat in others: The role of body morphology
Source: PLoS One. 2021 Apr 8;16(4):e0249782. doi: 10.1371/journal.pone.0249782 (PMC8031394; doi:10.1371/journal.pone.0249782)
Supplement: S3 Table — (DOCX) [file pone.0249782.s003.docx]

**S3 Table.** **Breakdown of the dimensions of the Daz human male body stimuli (in centimetres), varying by 7 levels of portliness.**

|  | **Below Knee Circ.** | **Ankle Circ.** | **Knee to Ankle** | **Inseam** | **Thigh Circ.** | **Crotch to Knee** | **Low Hip Circ.** | **Wrist Circ.** | **Elbow to Wrist** | **Forearm Circ.** | **Shoulder to Elbow** | **Bicep Circ.** | **Shoulder to Wrist** | **Bust Circ.** | **Waist Circ.** | **Waist to Thigh** | **Shoulder to Shoulder** | **Thigh to Floor** | **Collar Bone Neck to Floor** |
| --- | --- | --- | --- | --- | --- | --- | --- | --- | --- | --- | --- | --- | --- | --- | --- | --- | --- | --- | --- |
| **Port 1** | 33.49 | 23.19 | 42.60 | 78.04 | 56.31 | 35.44 | 93.58 | 17.78 | 26.35 | 27.68 | 30.57 | 31.70 | 56.92 | 101.93 | 83.24 | 23.53 | 37.22 | 81.14 | 152.21 |
| **Port 2** | 34.95 | 23.55 | 42.49 | 77.96 | 58.48 | 35.47 | 96.94 | 18.02 | 26.36 | 28.27 | 30.57 | 32.67 | 56.91 | 104.49 | 87.46 | 23.55 | 37.03 | 80.90 | 152.27 |
| **Port 3** | 36.42 | 23.91 | 42.38 | 77.88 | 60.65 | 35.50 | 100.29 | 18.26 | 26.36 | 28.85 | 30.57 | 33.67 | 56.94 | 107.06 | 91.69 | 23.58 | 36.83 | 80.67 | 152.34 |
| **Port 4** | 37.90 | 24.27 | 42.27 | 77.80 | 62.84 | 35.53 | 103.65 | 18.50 | 26.37 | 29.44 | 30.57 | 34.69 | 56.95 | 109.65 | 95.93 | 23.63 | 36.64 | 80.43 | 152.41 |
| **Port 5** | 39.38 | 24.63 | 42.16 | 77.73 | 65.03 | 35.57 | 107.00 | 18.74 | 26.38 | 30.04 | 30.57 | 35.73 | 56.96 | 112.25 | 100.19 | 23.68 | 26.47 | 80.19 | 152.48 |
| **Port 6** | 40.86 | 25.00 | 42.05 | 77.66 | 67.23 | 35.61 | 110.34 | 18.98 | 26.40 | 30.63 | 30.57 | 36.80 | 56.97 | 114.85 | 104.47 | 23.73 | 36.32 | 79.95 | 152.55 |
| **Port 7** | 42.35 | 25.38 | 41.95 | 77.60 | 69.44 | 35.65 | 113.68 | 19.22 | 26.41 | 31.23 | 30.56 | 37.88 | 56.98 | 117.45 | 108.77 | 23.79 | 36.19 | 79.72 | 152.62 |
