# Supplementary material for: Perceiving threat in others: The role of body morphology
Source: PLoS One. 2021 Apr 8;16(4):e0249782. doi: 10.1371/journal.pone.0249782 (PMC8031394; doi:10.1371/journal.pone.0249782)
Supplement: S4 Table — (DOCX) [file pone.0249782.s004.docx]

**S4 Table.** **Breakdown of the total change in centimetres of the Daz human male body stimuli having undergone transformations of musculature, emaciation and portliness.**

|  | **Musculature** | **Emaciation** | **Portliness** |
| --- | --- | --- | --- |
| **Level 1** | 0 | 0 | 0 |
| **Level 2** | 9.96 | -6.89 | 15.42 |
| **Level 3** | 20.14 | -13.65 | 30.93 |
| **Level 4** | 30.23 | -20.33 | 46.55 |
| **Level 5** | 41.05 | -26.92 | 52.26 |
| **Level 6** | 51.72 | -33.46 | 78.05 |
| **Level 7** | 62.58 | -39.74 | 93.95 |
