# Supplementary material for: Perceiving threat in others: The role of body morphology
Source: PLoS One. 2021 Apr 8;16(4):e0249782. doi: 10.1371/journal.pone.0249782 (PMC8031394; doi:10.1371/journal.pone.0249782)
Supplement: S11 Table — Model 1 regresses PT onto change in musculature only. Model 2 regresses PT onto change in portliness, PA, age, gender, education and order of block presentation. Model 3 additionally includes height and weight. The best-fitting model (Model 1) was identified using the Akaike Information Criterion (AIC), the results of which were reported in the main paper. (DOCX) [file pone.0249782.s011.docx]

For each of the models reported in S5 – S12 Tables, we used mixed-effects ordered logistic regressions to estimate the influence of the different independent variables on perceptions of threat, assuming a random effect across participants, with robust standard errors clustered at the participant level to account for intra-participant correlation.

Threshold for significance (alpha) was set at a value of .05. The assumption of lack of multicollinearity was met for all variables. The requirement of proportionality of odds was relaxed for variables that failed standard checks of this assumption (flagged in Tables S5-S8 in the Supplemental Material). For these instances, we re-ran the analyses with generalised ordered logistic regressions. These did not qualitatively affect or change the findings. Therefore, we simply report the ORs from the standard ordered logits.

**S11 Table. Odds ratios from ordered logit models predicting perceived threat in the musculature-varying body stimuli (Experiment 2).** Model 1 regresses PT onto change in musculature only. Model 2 regresses PT onto change in portliness, PA, age, gender, education and order of block presentation. Model 3 additionally includes height and weight. The best-fitting model (Model 1) was identified using the Akaike Information Criterion (AIC), the results of which were reported in the main paper.

|  | Model 1† | Model 2 | Model 3 |
| --- | --- | --- | --- |
| Musculature/Δcm | **1.05*****  [1.04, 1.06] | **1.05*****  [1.04, 1.06] | **1.05*****  [1.04, 1.06] |
| Perc. Attractiveness‡ |  | 0.94  [0.67, 1.34] | 0.94  [0.67, 0.33] |
| Age |  | 1.03  [0.97, 1.09] | 1.03  [0.98, 1.09] |
| Gender *(Ref: Male)* |  | 1.25  [0.27, 5.84] | 0.99  [0.17, 5.77] |
| Education *(Ref: High School)*‡ |  |  |  |
| 2 |  | 1.86  [0.43, 8.14] | 1.66  [0.38, 7.29] |
| 3 |  | 3.15  [0.17, 58.10] | 2.17  [0.15, 31.45] |
| 4 |  | 2.55  [0.13, 50.10] | 1.68  [0.08, 37.37] |
| Order |  | 0.76  [0.21, 2.79] | 0.79  [0.22, 2.89] |
| Height |  |  | 1.88  [0.36, 9.90] |
| Weight |  |  | 0.29  [0.06, 1.28] |
| Obs. | 1050 | 1050 | 1050 |
| Participants  AIC | 150  1884.00 | 150  1895.32 | 150  1896.12 |

**p* < .05, ***p* < .01; ****p* < .001;

† Model reported in main paper

‡The proportionality of odds assumption was relaxed for these predictors.

*Note.* 95% Confidence Intervals in brackets.
