# Supplementary material for: Perceiving threat in others: The role of body morphology
Source: PLoS One. 2021 Apr 8;16(4):e0249782. doi: 10.1371/journal.pone.0249782 (PMC8031394; doi:10.1371/journal.pone.0249782)
Supplement: S13 Table — (DOCX) [file pone.0249782.s013.docx]

**S13 Table. Odds ratios indicating the effects of facial threat and musculature on compound perceived threat at the varying cuts of musculature and facial threat respectively.**

| **Odds Ratio for Effect of Facial Dimension** | | **Odds Ratio for Effect of Musculature** | |
| --- | --- | --- | --- |
| **Muscle 1** | 2.24 | **-3 Facial Threat** | 1.51 |
| **Muscle 2** | 2.04 | **-2 Facial Threat** | 1.41 |
| **Muscle 3** | 1.93 | **-1 Facial Threat** | 1.42 |
| **Muscle 4** | 1.97 | **0 Facial Threat** | 1.30 |
| **Muscle 5** | 1.99 | **+1 Facial Threat** | 1.29 |
| **Muscle 6** | 1.73 | **+2 Facial Threat** | 1.24 |
| **Muscle 7** | 1.90 | **+3 Facial Threat** | 1.29 |

Note: All *p*s for cut models are less than 0.001
