# Supplementary material for: Perceiving threat in others: The role of body morphology
Source: PLoS One. 2021 Apr 8;16(4):e0249782. doi: 10.1371/journal.pone.0249782 (PMC8031394; doi:10.1371/journal.pone.0249782)
Supplement: Attachment — Submitted filename: Response to Reviewers .docx [file pone.0249782.s014.docx]

**Response to the editor**

We would like to thank the Editor and reviewers for their constructive suggestions on our manuscript entitled “Perceiving threat in others: the role of body morphology”. Based on their comments we have revised our manuscript accordingly. The most substantial revisions relate to deviations from the pre-registered protocol in Experiment 1, as highlighted by both the Editor and Reviewer 2.

The manuscript deviated from the pre-registration in three main ways. First, the pre-registration contained a greater number of individual hypotheses. The submitted manuscript had collapsed these into broader, more concise hypotheses. These have since been restored in the revised manuscript to the original number of hypotheses to match the pre-registration. Second, the manuscript described an a priori G*Power analysis to justify the sample size of Experiment 1. This was done following the publishing of the pre-registration, and so should not have been included. This has now been replaced by a post-hoc estimation of achieved power. Third, the pre-registered protocol had outlined the use of linear models to analyse perceived threat in the face and body stimuli. We later decided that, as ordinal scales had been used to record perceived threat, it would be more apt to employ a model used specifically for this type of data. This has now been clarified in the revised manuscript, where a detailed justification for this change is included in the new “Data Analysis” subsection found at the conclusion to the Methods section.

In addition, as per request, Figure 3, which depicted examples of the compound stimuli, has been removed such as to comply with the Creative Commons Attribution License (CCAL), CC BY 4.0. Therefore, the numbering for each following figure has been updated. The paper also now more closely follows PLOS ONE’s style requirements. Below we address these, and all other concerns raised by the three reviewers.

**Response to reviewers**

We thank the reviewers for reviewing our manuscript “Perceiving threat in others: the role of body morphology” and for their constructive comments. Below we include a point by point reply to the issues raised by each reviewer.

**Reviewer 1**

***The one minor suggestion I have is that I found some of the Figures confusing, in particular, Figure 5 and Figure 6. For instance, could some colour be introduced in Figure 5 to help distinguish between the faces? Also, in Figure 6, it is unclear to me what is on the y-axis.***

- We agree that Figures 5 and 6 (now Figures 4 and 5 in the updated manuscript due to the removal of Figure 3) could have been made clearer. These have been revised accordingly and Figure 5 now includes different colours to distinguish the varying levels of facial threat and musculature of the stimuli. We have improved the y-axis label of Figure 6 to increase clarity.

**Reviewer 2**

***I found the hypotheses of Experiment 1 to be quite light – in particular, the hypotheses in the manuscript did not provide clear predictions about the different manipulations of body morphology that the authors actually used. Furthermore, these hypotheses appear to be different from the pre-registered hypotheses on the OSF registry, where the hypotheses and predictions are much clearer and more explicitly spelt out. I would encourage the authors to stick to the hypotheses as laid out in their preregistration – I also felt that these would follow the structure of the results section more intuitively.***

- We originally condensed the hypotheses used in the pre-registration to improve the readability of the manuscript. However, this did leave the individual hypotheses lighter in detail and not as clearly structured as they had originally appeared in the pre-registration. In the revised manuscript, each body manipulation now has its own separate numbered hypothesis with justification, matching the pre-registration (see pages 5/6 of the revised manuscript). These numbered hypotheses are now also directly referred to in appropriate subsections of the Results.

Also, the first pre-registered hypothesis of Experiment 2 (concerning the ICCs for perceived threat) had not been explicitly stated in the text. This is now clearly stated (see page 14).

***The authors report an a priori G*Power analysis as justification of their sample size in Experiment 1 on p.5, but no such analysis is reported in the preregistration document, where the sample size is instead justified on the basis of previous research.***

- This was an error on our part. This a priori G*Power analysis was done following the publication of the pre-registration, and so should not have been included in this pre-registered paper. This has now been replaced by a post-hoc estimation of achieved power (see page 6).

***The authors have completely omitted to report the sample size of Experiment 2 (p.13).***

- We thank the reviewer for pointing this out. This has been amended in the revised manuscript (page 15).

***It is not clear to me why the demographic data were collected. Participant height and BMI are explained (although glossed over in the results section as they consistently yield null results) but it is not clear why (in particular) age or education would be considered so important in the current study, and consequently it is difficult to know how to interpret the observed relationship between having a bachelors degree and finding muscular bodies threatening.***

- We thank the reviewer for pointing this out. We agree that justification of the inclusion of the age and education variables was limited and lacked further discussion. As these were not key variables of interest, but rather control variables, the original manuscript did not go into much detail. The revised manuscript addresses this at the conclusion of the Introduction by more clearly illustrating why they were included as controls (page 6). Furthermore, the Discussion section now includes a paragraph that briefly discusses the observed relationships between age, education and perceived threat (page 26).

***Conversely, if the authors anticipated an interaction of attractiveness and participant gender, I am curious as to why information such as sexual orientation was not also gathered.***

- We thank the reviewer for this comment. This is a valid limitation, which is now noted in the Discussion of the revised manuscript (page 25).

Minor content comments:

1) ***P.11 – In the results of portliness, there are two instances where the authors instead refer to “the level of emaciation”***

- Thank you, this has been fixed.

2) ***Regarding H6, it is not clear what the threat dimension of the face stimuli being ambiguous means. Specify that (presumably) this ambiguity refers to these faces being rated neutral.***

- Thank you, more detail has now been added in the text to make this clearer (page 14).

3) ***P.15 – I wonder if the authors would care to comment on the lower consensus on attractiveness ratings in Experiment 2 (ICC=.49) for the body-only stimuli relative to the same stimuli in Experiment 1 (ICC=.80).***

- Thank you, we agree that this fall in consensus is most likely attributable to their presentation in the same block as the compound stimuli. This is now addressed in the text (page 18).

4) ***P.16 – No analysis of other variables (attractiveness, age, gender, etc.) is reported for the body-only stimuli of Experiment 2. If this is because this analysis yielded null results, these should still be reported.***

- In the original manuscript, it was unclear as to how the models reported in the text were selected. We have now clarified that these were selected using the Akaike Information Criterion (AIC). For the body-only stimuli in Experiment 2, the best-fitting model was the initial model, in which threat was regressed onto only the changes in musculature. The other variables had no significant effect, and disimproved the fit of the model, and so were not reported. This has now been made clear in the text (page 19)

5) ***Please label all axes of figures with relevant information – these should be interpretable to readers without having to mine the text (e.g. Fig 6, y axis)***

- Thank you, this has been changed.

6) ***P.22 – The suggestion of using VR to display life-sized human stimuli is a nice idea, but you could also take this further by manipulating the size and body morphology of the participants’ own body avatar in VR to test additional predictions.***

- This is a really nice suggestion, thank you. We have added it to the discussion (page 26).

Minor stylistic comments:

***P.4 – When reporting the results of previous studies in text using a numbered citation system, consider writing out the names of authors in the text; e.g. “For example, Schvey et al. [26] found that, in mock trials, male jurors…” instead of “For example, [26] found that, in mock trials, male jurors…”***

- Thank you, this has been changed.

***While they can be helpful when writing, abbreviations like PT and PA rarely actually help readers understand, particularly when these could be refer to simply as “threat” and “attractiveness”, which would be much easier to read***

- These abbreviations have now been largely removed, aside for some uses in figure axes.

**Reviewer 3**

***The theme of this paper "threatening perception" seems to be based on the trait perception, but how does this differ from emotion perception? More profound reviews for previous studies and discussion for the current results would be better.***

- Thank you, the difference between emotion and trait perception is a key distinction for the current paper. This distinction has now been highlighted and clarified in the Introduction (see page 3 of the revised manuscript). Also, a more expanded paragraph on the link between emotion and trait perception has been added to the discussion (see page 24).

***In Fig 3, the size of the face itself seems to change depending on the parameters, which may need to be considered***

- The sizes of the faces in the compounds were slightly adjusted to match the size of the paired bodies more naturally. Although this may have introduced some slight variability in how the faces were perceived, it was decided that this was preferable to presenting noticeably incongruent face and body combinations. This adjustment of face size has now been clarified in the compound stimuli subsection of the Methods section of Experiment 2 (page 16).

***The results reported indicate that age also affected the results. It is recommended to add a discussion of this point.***

- We thank the reviewer for their comment. As also noted by Reviewer 2, the analyses of the effects of age and education was lacking further discussion. The revised manuscript addresses this, with the Discussion section now including a paragraph that briefly discusses the observed relationships between age, education and perceived threat (page 26).

***What is the role of the face in body morphology for threatening perception? It would be better to organize the discussion a little more to clarify your argument.***

- We understand that we may not have fully clarified the primary role played by facial information in the perception of threat. Although we have shown that bodies play a significant role, regardless of facial information, it does seem that the judgment is still primarily driven by the face. This has now been made clear in the opening of the Discussion (page 23).

***The lack of structured descriptions in the Methods and Results section gave the impression that the data were difficult and costly to interpret. Some of the analysis methods (e.g. intraclass correlation coefficients and logistic regression analysis) are common to both Experiment 1 and 2. Then, it would be better to describe them in a separate section "Data Analysis". Also, for the logistic regression analysis, it would be desirable to have more detailed descriptions of the independent and dependent variables as well. They are likely to be necessary information to ensure reproducibility and to judge whether the statistical analysis is being appropriate.***

- We appreciate that the results may be difficult to follow because we have a number of analyses to present. However, both Reviewers 1 & 2 indicated that they thought the results and analyses were particularly clear, so we have tried to clarify sections without substantially changing the structure. To achieve this, we followed the reviewer’s suggestion and have added a “Data Analysis” subsection to the end of each Methods section (pages 9/17). This outlines the analysis steps due to follow in each subsequent Results section. We also now include details of the ICC and regression analyses that were previously found in the Results section.

Minor points:

1) ***There were several areas where the literature was not properly cited. (e.g. the Introduction section [26]-[28] in p.4) Please recheck the citation format.***

- Thank you, this has been fixed.

2) ***Recheck the captions in the tables S5-S8 to make sure they are correct. (AIC value is smaller in other Models.)***

- The reported models are from the best-fitting models that are also fully-powered. These models do not include height/BMI, as 31 participants opted not to disclose this information.

3) ***Tables and figures are requested to be placed in appropriate and effective places in your paper. It would be unfavourable to list them all in one place***

- We thank the reviewer for the suggestion. Figures now appear in the manuscript at the conclusion of the paragraph in which they were first mentioned. Although regression tables could have been included in the manuscript itself, we decided to omit them from the main text. We felt that plotting out the relationships visually would communicate the relationships between the key variables more clearly.

4) ***The way "H1" might mean "Hypothesis 1"(e.g. p.5), however, it may not be a universal usage. Other abbreviations such as IV was first mentioned (p.9), so it would be better to pay more attention to the use of abbreviations.***

- Thank you, all abbreviations have now been clarified at their first use in the text.

5) ***How many participants joined in Experiment 2 finally?***

- We thank the reviewer for pointing this omission. This has been amended in the revised manuscript (page 15).

6) ***It would be better to elaborate more on the relationship between the number of stimuli in Experiment 2 to the number of trials? (Does it include the number of distractions?)***

- The exact number of trials for experimental, distractor, body and compound stimuli in Experiment 2 is now explicitly stated in the text (page 17).

7) ***It would be better to describe the dataset of the face stimuli in more detail. What other parameters are in the dataset besides "threatening"? It would be better to write in a way that the reader can understand a summary of the presented stimuli without referencing the research by Todorov et al. (2013) [33].***

- More detail has now been added to the Methods section to more fully describe the method used by Todorov to produce the CG face stimuli (page 7).
