# Supplementary material for: Perceiving threat in others: The role of body morphology
Source: PLoS One. 2021 Apr 8;16(4):e0249782. doi: 10.1371/journal.pone.0249782 (PMC8031394; doi:10.1371/journal.pone.0249782)
Supplement: Attachment — Submitted filename: Response to Reviewers.docx [file pone.0249782.s015.docx]

**Response to the editor**

We would like to thank the Editor and reviewers for their constructive suggestions on our manuscript entitled “Perceiving threat in others: the role of body morphology”. Based on their comments we have revised our manuscript accordingly. The main revisions relate to queries by Reviewer 3 regarding expansions on the holistic processing hypothesis and additional detail on the distinction between emotion and trait perception. Below we address these, and all remaining concerns raised by the reviewers.

**Response to reviewers**

We thank the reviewers for reviewing our manuscript “Perceiving threat in others: the role of body morphology” and for their constructive comments. Below we include a point by point reply to the issues raised by each reviewer.

**Reviewer 2**

***Regarding the power analysis, I agree that the use of an a priori analysis is not appropriate if it was performed following data collection. Given that the authors do report an a priori justification for their sample size in both their preregistration and manuscript, I would drop this post-hoc calculation. If a power calculation is required, I would recommend looking into simulation-based approaches.***

- We thank the reviewer for this comment. We agree that post-hoc power calculations can often be more problematic than helpful. Also, as the reviewer has mentioned, we did originally report an a priori justification for our sample size in both the preregistration and manuscript.

Therefore, as suggested, we have removed the post-hoc power calculations from the manuscript.

***p.9 – when first defining abbreviations, spell out the full abbreviation (i.e. ‘intraclass correlation coefficient (ICC)’ rather than just correlation coefficient).***

- Thank you, this has now been fixed (page 9).

***As far as I can tell from the supplementary material, the AIC was used to select the best fitting model in both experiments, but is only mentioned in Experiment 2 because it selected the simpler model over the full. If this model comparison was conducted, it should be reported in the main text for Experiment 1 as well.***

- Thank you, the use of the AIC is now reported in the Data Analysis sections of the Methodology for both Experiments 1 (page 9) and Experiment 2 (page (17)

**Reviewer 3**

***Thank you for adding the description of emotion perception and trait perception. It helped me understand the points. However, there is another ambiguous term: personality. I am curious about the difference between personality and trait in the manuscript. It would be helpful to add some descriptions of personality and traits.***

- Thank you, we now appreciate that we have used the terms “personality” and “trait” somewhat interchangeably. When we have referred to “trait perception”, the full term we are referring to is “personality trait perception”. We have now clarified this with use of the full term in the early parts of the introduction section (pages 3/4).

***Furthermore, which process is more similar to "perceiving threat": "trait perception" or "emotion perception"? I've regarded "perceiving threat", examined in this study, as a part of judging the traits of a person presented as a stimulus. However, now I feel that it contains both aspects: trait perception and emotion perception. In the last paragraph on p.4, the authors argued that perceiving threat might influence the judge of traits or personality. Therefore, it would be more persuasive if the authors clarify the position on whether they regard “perceiving threat” as more like “trait perception” or “emotion perception”.***

- We thank the reviewer for this comment. We agree that this is a very important distinction to draw. An emotional signal can indeed influence how a person is perceived. However, emotions tend to be transient in nature. Hence, the goal of the current paper is to go beyond inferences that may be reliant on, or driven by, perceived emotion. Therefore, for the purposes of the current paper, we are focusing on threat as a perceived trait, rather than as a perceived emotion. We have further specified the distinction between emotion and trait perception, and more clearly stated our goal of investigating personality trait inferences, in the manuscript (page 3).

***- In the last hypothesis on p5, the authors refer to the holistic processing of faces and bodies and hypothesized that faces and bodies would play significant roles respectively. This hypothesis sounds very general, and it would be good to add some more description of the holistic person-perception hypothesis given in previous works. This is also mentioned in the Discussion section (p.26) and would be a key point in the manuscript. For a better understanding of the authors’ hypothesis about perceiving threat, it would be useful to add more explanations of the reference [10].***

- We thank the reviewer for their comment. We have provided additional information about the nature of the holistic processing hypothesis forwarded by Aviezer et al. [10], along with more detail about the experiments put forth in their paper as evidence for the hypothesis (page 3).

***Am I correct in understanding that the final number of participants was 150 for experiment 1 (p.7), of which 87 were women, and 100 for experiment 2, of which 74 were women (p.16-17)? It would be clear for readers if the authors clarify the total number of participants.***

- Thank you, this has now been clarified in the manuscript.

***It is a trivial matter, but in hypothesis H5 and H6, the order was Portliness (H5) and Emaciation (H6) (p.5). Therefore, it would be helpful if the order of the results (p.13-15) match with the order written in the hypothesis, for a smoother understanding.***

- Thank you for this helpful suggestion, the hypotheses have now been reordered to match the layout of the results section.

***Regarding the last paragraph in section 3.2.2.3 on p. 22, the authors argue that contrary to H7 and H8, there was no correlation between the musculature or facial dimension and the compound stimuli ratings. However, I'm not sure why this conclusion was drawn. For example, for H7, I thought it needed to compare the correlation coefficients when the face was ambiguous (i.e., when the face dimension level was about 1-3) and when the face dimension level was other levels. However, only overall correlation coefficients have been reported, and is it possible to draw such a conclusion? I apologize if I misunderstand the results.***

- Thank you, we see now that this was not very clearly worded. We did not mean to suggest that no correlations were found between perceived threat in the face/body stimuli and compound stimuli. This was not the case, as threat in the compounds was significantly correlated with all levels of face-only and body-only threat. Rather, no clear pattern in correlation by level of musculature or facial dimension emerged, which was contrary to H7 and H8. We have now clarified this distinction in the manuscript (page 21).

***As I mentioned above, holistic processing is the key to the authors’ hypothesis and interpretation of the results, but the explanation may be insufficient. So, I cannot fully understand how the results supported the holistic person-perception hypothesis (p.26). It would be helpful if the authors add more explanations of the holistic person-perception hypothesis in the Introduction section and rephrased it shortly in the Discussion section***

- As suggested, more information about the hypothesis has been provided in the Introduction, with an additional sentence in the Discussion (page 24) detailing how the perception of the compounds diverged from a simple linear combination of the threat perceived in the face and body stimuli.
